# Simulation-based education for teaching aggression management skills to health care providers in the acute health care setting: a systematic review protocol

**DOI:** 10.1186/s13643-020-01466-8

**Published:** 2020-09-04

**Authors:** Marijke Mitchell, Charmaine Bernie, Fiona Newall, Katrina Williams

**Affiliations:** 1 Neurodevelopment & Disability, Royal Children’s Hospital, 50 Flemington Road, Parkville, Victoria 3052 Australia; 2 Department of Allied Health, Royal Children’s Hospital, 50 Flemington Road, Parkville, Victoria 3052 Australia; 3 Nursing Research, Nursing Education, Royal Children’s Hospital, 50 Flemington Road, Parkville, Victoria 3052 Australia; 4 Department of Paediatrics, The University of Melbourne, 50 Flemington Road, Parkville, Victoria 3052 Australia; 5 Department of Nursing, The University of Melbourne, 50 Flemington Road, Parkville, Victoria 3052 Australia; 6 Murdoch Children’s Research Institute, 50 Flemington Road, Parkville, Victoria 3052 Australia; 7 Department of Paediatrics, Education and Research, Monash Children’s Hospital, Monash University, 246 Clayton Road, Clayton, Victoria 3168 Australia

**Keywords:** Systematic review protocol, Simulation training, Aggression, Health personnel

## Abstract

**Background:**

Clinical aggression episodes, that is aggression and externalising behaviours that create risk, in acute care hospitals are increasing. Acute care staff are often not confident or trained in the management of aggression. Various aggression management training formats have been described in practice including face to face training, written learning resources, web- and media-based training resources, and simulation training. The aim of this systematic review is to assess whether simulation-based training is effective in increasing de-escalation knowledge, skills, and behaviour of staff working in the acute care setting.

**Methods:**

We designed and registered a study protocol for a systematic review of studies evaluating simulation-based training for the management of patients with aggression. We will include randomised controlled trials, non-randomised controlled trials, quasi-experimental studies, and observational studies including health care professionals and trainees in acute health care settings. Comprehensive searches will be conducted in the following databases (from January 1980 onwards): PubMed, MEDLINE, PsycINFO, CINAHL, and the Cochrane Library. The reference lists of selected studies, trial registers, and leading journals will also be searched. Two reviewers will independently screen all citations, full-text articles, and abstract data. Potential discrepancies will be resolved through discussion. The primary outcomes will include patient outcomes (e.g. frequency of clinical aggression), quality of care (e.g. frequency of emergency situations, physical/chemical/mechanical restraint), and adverse effects (e.g. patient/family complaints, patient harms, staff harms). Secondary outcomes will include workplace resource use, health care provider-related outcomes, knowledge (de-escalation techniques), performance, attitudes, and satisfaction. The study methodological risk of bias will be appraised using appropriate tools. A narrative synthesis will be performed for included studies. If feasible, we will conduct random-effects meta-analysis of data. Additional analyses will be conducted to explore the potential sources of heterogeneity (e.g. participant characteristics, interventions, and follow-up).

**Discussion:**

This systematic review will identify, evaluate, and integrate the evidence on simulation-based training programmes for acute care health professionals on managing clinical aggression. The results of this study will inform the implementation of effective training strategies. Implications for future research will be discussed.

**Systematic review registration:**

PROSPERO registration number CRD42020151002

## Background

### Rationale

Aggression initiated by patients towards themselves, staff, and parents, carers, or family members is increasing in frequency in acute care hospitals worldwide [1–8]. In some countries, the health industry has been found to be one of the most violent industries [9, 10]. Also, of concern are other externalising behaviours, like wandering, that place a person at risk. Some studies have shown that aggression minimisation programmes have been found to improve staff knowledge, confidence, skills, and attitudes towards dealing with aggression in the workplace [11–18]. Increasing staff confidence and skill may result in less episodes of aggression and improved patient experiences.

Increase in the number of episodes of clinical aggression is not unique to the adult setting. Paediatric hospitals are reporting increased levels of aggression [19–21] with over 1600 events at one Australian paediatric tertiary hospital reported in 1 year [22]. Aggression, destruction of property, wandering, and self-harm can result in injury to self and others [23–28]. Hyperactive, impulsive behaviour in hospitalised children with autism spectrum disorder (ASD) can result in absconding and further safety issues [29]. Externalising behaviours are common in children with ASD when exposed to hospital stressors and can result in difficult or delayed treatment, increased anxiety for the parent and child, prolonged procedure times, increased health care costs, and poorer health outcomes [30–33]. In the adult setting, patients most likely to exhibit aggressive behaviours are those diagnosed with psychiatric or personality disorders, dementia, acute confusion, or drug-related problems [34]. In the paediatric setting, approximately 50% of aggressive episodes have been noted to be initiated by children with a neurodisability [22].

Staff who are exposed to aggression are subject to both the physical abuse and subsequent emotional effects [13]. Continued exposure to aggression can reflect negatively on staff recruitment and retention [1]. For staff, high levels of stress and burnout have been documented when caring for individuals with aggressive behaviours [35, 36]. Nurses in acute care settings report feelings of emotional pain, anger, and increased sick leave as a result of exposure to aggression [37].

Simulation-based education has been used in the acute health care setting to train staff to manage acute cardio-respiratory patient deterioration, resuscitation, and improve interpersonal and team communication. Simulation-based training for communication skills has been shown to be an effective means of teaching communication skills [38] and improving patient safety [39, 40]. Kaplonyi et al. [38] published a systematic review on the impact of simulated patients on health care learners’ communication skills. This is the first review to examine if simulated patient-based communication skills training improves learner-patient communication. This review included a diverse range of communication training interventions including breaking bad news, disclosing error, working with patients with impaired cognitive states, smoking cessation, and counselling. This review demonstrated that the use of simulated patients is an effective means of teaching communication skills however was not able to demonstrate economic benefit for this type of training over other formats. The results were categorised against outcome measures and then synthesised against the levels of Kirkpatrick’s Hierarchy of Learning [41]. The review did not include studies where the primary focus was the use of simulation training for aggression management.

Simulation training for managing aggressive behaviours provides an opportunity to practice de-escalation skills in a high-fidelity situation. Broadly, simulation training allows participants to develop or enhance their knowledge and skills, and analyse and respond to realistic situations in a simulated clinical setting, without patient risk, and may accelerate the learning process [42]. The use of simulated patients as an interactive means of teaching and applying and practising communication skills is gaining momentum [38]. Learners report the use of simulated patients in training to be more beneficial than didactic lectures and other written learning resources. In particular, the feedback provided about their performance is reported to contribute to improvement in their individual specific skill development [38]. The major barrier to implementation of simulation training for acute care staff in the management of clinical aggression is cost. This form of training is time and resource intensive, and unless proven to be highly efficacious with a high return on investment, hospital administrators will not approve its use over more traditional and less costly forms of training.

The focus in mental health facilities is on optimising mental health for clients and residents with care provided by trained mental health staff with expertise in managing aggressive behaviour. Aggression management training programmes in the psychiatric and mental health settings have been reviewed and evaluated [15] some of which include the use of simulated patients. However, the results cannot be generalised to the acute setting or further extrapolated to the paediatric setting due to the different focus of care and differences in staff baseline mental health expertise [7, 14].

In acute care settings, care is primarily focussed on the management of physical deterioration rather than mental deterioration with clinicians highly trained in acute disease management and resuscitation. Considering the frequency of aggressive or other externalising behaviours in acute care hospitals and the potential threat to the safety of patients, families, and staff, it is important to understand if aggression management training utilising simulated patients is an effective training format for acute care clinicians to increase knowledge, skills, and behaviour. This sophisticated style of training may also be effective in reducing the frequency and severity of aggressive incidents.

Our review, which provides a focussed examination of the use of simulated patient-based training to manage clinical aggression in the acute care setting, will make a valuable contribution to what is known and not known about this increasingly common and complex area of acute health care delivery.

The objective of this study will be to assess the effectiveness of simulation-based education for teaching aggression management skills to health care providers in the acute care setting.

The specific review questions will be as follows:

1. Are participants able to apply their learnings from the simulation-based education to the work setting and reduce episodes of aggression and associated clinical interventions and sequelae?
2. Does simulation training increase participants’ knowledge, skills, and confidence in managing clinical aggression?
3. Is simulation training an acceptable format of training for teaching management of clinical aggression to acute care health professionals?

## Methods

The present protocol has been registered within PROSPERO (registration ID: CRD42020151002). The present study protocol is being reported in accordance with the reporting guidance provided in the Preferred Reporting Items for Systematic Reviews and Meta-Analyses Protocols (PRISMA-P) statement [43] (see PRISMA-P checklist in Additional file 1).

### Criteria for considering studies in this review

Studies will be selected according to the criteria outlined below.

### Study designs

We will include randomised controlled trials (RCTs, including cluster RCTs), controlled non-randomised controlled trials, quasi-experimental studies (including controlled before-after studies, interrupted time series studies), and observational studies (including repeated measures studies/crossover studies, cohort studies). Included study designs have been selected to ensure that outcomes are compared over time or with a group who have been offered no intervention or an alternative intervention.

We will exclude case reports, case series, case control studies, commentaries, editorials, opinions or purely descriptive studies, review articles, and conference proceedings.

### Participants

We will include all studies of health care professionals in acute hospital settings who receive simulation-based training to manage patients with aggression. Participants may include nurses, doctors, and allied health professionals, and these professionals will need to make up at least 70% of participants. Studies including trainee health professionals who receive simulation-based training in a university setting as part of their undergraduate training will also be included.

### Interventions

We will include all simulation-based education using people as simulated patients provided to health care professionals or health care trainees to teach prevention and management of aggression.

We will include all forms of simulation-based education which may include descriptive terms such as standardised patient, simulated patient/person, standardised patient simulation, and role play. Additional terms that may be used in the description of the training approach include simulationist, role player, actor, confederate, embedded participant, and simulation fidelity. The Agency for Health care Research and Quality (AHRQ) in partnership with the Society for Simulation in Healthcare, and its many affiliates, compiled a comprehensive Healthcare Simulation Dictionary which defines terms used in simulation health care. The common simulation terms which relate to this systematic review are defined in Additional File 2 [44].

Aggressive and externalising behaviours that place individuals at risk are described in the literature in many ways, as listed in Additional File 3.

We will exclude studies that use simulation for non-educational purposes or where the primary objective is not related to managing aggression. Any studies that do not use simulation training in real time using a live person as the patient, for example, online, video recorded, or paper-based simulation; virtual reality simulation; manikin-based simulation; or task trainer simulation, will not be included. Studies in which the participants are not actively involved in the simulation training will also be excluded.

### Comparators

We will include studies comparing simulation to the following: (1) no training, (2) other forms of training, and (3) baseline measures when no comparison group is available. We will also include studies in which simulation was added to other training common to all participants.

We will use the training outcome hierarchy presented by Kirkpatrick [41] to characterise the level of evaluation as reaction (satisfaction), learning, behaviour, or results (patient outcomes). This hierarchy (Table 1) is considered by the World Health Organization (WHO) to be the standard reference for evaluation of training in the health context [45].

**Table 1:** Kirkpatrick framework [41]

Level	Evaluation	Evaluation description
1	Reaction	Measures participants’ reaction to the training and satisfaction
2	Learning	Measures to what degree participants acquire the intended knowledge, skills, attitudes, confidence, and commitment based on their participation
3	Behaviour	Measures to what degree participants apply their learnings from the training to the work setting
4	Results	To what degree targeted outcomes, including change in professional practices and benefits to patients, occur as a result of the training

## Outcomes and prioritisation

### Primary outcomes

The primary outcomes, as outlined in Table 2, will be the number of incidences of aggression; frequency of behavioural emergency situations; frequency of use of chemical, physical, and mechanical restraint; incidences of patient and staff harm; and patient complaints up to 1 year following the simulation training. We have chosen these outcomes as they will provide a clinical picture of the amount of clinical aggression reported in acute care settings and the effect staff training has on the use of interventions to manage and reduce clinical aggression and maintain patient and staff safety. We anticipate including the primary outcomes in a summary of findings table. A more detailed definition of outcomes has been included in Additional File 4.

**Table 2:** Primary outcome measures

Primary outcomes	Outcome measures	Kirkpatrick level
Patient outcomes	- Frequency of clinical aggression	4
Quality of care	- Frequency/numbers of behavioural emergency situations - Physical restraint - Chemical restraint - Mechanical restraint	4
Adverse effects or harms	- Patient/family complaints - Patient harm - Staff harm	4

### Secondary outcomes

In order to identify the components and conditions required to implement a quality simulation programme, we will also include secondary outcomes as described in Table 3. Studies will be included if they report data on any of the primary or secondary outcomes.

**Table 3:** Secondary outcome measures

Secondary outcomes	Outcome measures	Kirkpatrick level
Workplace resource use	- Activation of emergency response - Utilisation of skills taught in simulation	3
Health care provider	- Workload - Work morale - Stress - Burnout - Sick leave	3
Knowledge	- De-escalation techniques	2
Performance in test situation	- Self-reported confidence levels - **Observer rating of performance**	2
Attitudes	- Reactions to training	1
Satisfaction	- Satisfaction with training format - Authentic environment - Quality of the acting - Quality of the post-simulation debrief	1

The secondary outcomes can be divided into two groups. The first are health care provider outcomes in the workplace following the training: ability to activate the emergency response and utilisation of skills taught in simulation coupled with changes in reported workload, morale, stress levels, burnout, and sick leave following completion of the training as detailed in Table 3. The second are relating to reactions and learnings from the training including the following: reactions to the format, fidelity, quality of acting, and post-simulation debrief; use of de-escalation techniques within the training; and self-reported confidence levels post-training.

### Timing

Studies which evaluate the impact of the training immediately following completion and those that collect follow-up data evaluating the impact of the training up to 2 years following completion will be included.

### Setting

Only studies conducted in acute health care facilities and universities as part of health professional education will be included.

### Language

We will include all languages in the article search. Articles screened will be those reported in English, those where a minimum of the title and abstract are reported in English, and those which can be adequately translated using Google Translate (https://translate.google.com/). A list of possibly relevant titles in other languages will be provided as an appendix.

## Search methods for identification of studies

### Information sources and search strategy

The primary source of literature will be a structured search of electronic databases (from January 1980 onwards): PubMed/MEDLINE, MEDLINE (Ovid), Cochrane Library (Cochrane Central Register of Controlled Trials [CENTRAL], and Cochrane Database of Systematic Reviews), PsycINFO (Ovid), and CINAHL (Ebsco).

The secondary source of potentially relevant material will be a search of the grey or difficult to locate literature, including Google Scholar and clinical trial registers (such as World Health Organization trial search portal: http://apps.who.int/trialsearch, and ClinicalTrials.gov).

To ensure literature saturation, we will perform hand-searching of the reference lists of included studies, relevant reviews, or other relevant documents. Efforts will be made to contact authors of completed, ongoing studies, and in-press literature for information regarding additional studies, relevant material, or missing data.

In addition, the WHO International Clinical Trials Registry Platform Search Portal and ClinicalTrials.gov will be searched for ongoing or recently completed trials and PROSPERO will be searched for ongoing or recently completed systematic reviews. As relevant studies are identified, reviewers will check for additional relevant cited and citing articles.

The MEDLINE search strategy will be created by a Health Sciences Librarian with expertise in systematic review searching (PC) in conjunction with the corresponding author (MM). The main literature search will be peer-reviewed by a second health information specialist, not involved in the project, using the Peer Review of Electronic Search Strategies (PRESS) checklist [46]. The search will be based on Medical Subject Headings (MeSH), a broad range of terms and keywords related to the following: ‘simulation-training’, ‘communication’, ‘health care professionals’, and ‘aggression’. A draft search strategy for MEDLINE (Ovid) is provided in Additional file 5.

The search will be updated towards the end of the review to ensure relevant recent articles are included.

## Study records

### Data management

Literature search results will be uploaded to Endnote X9® and then copied to Covidence®, a web-based software platform which streamlines the production of systematic reviews and allows collaboration among reviewers during the study selection process. The authors will develop and test screening questions and forms based on the inclusion and exclusion criteria. Citation abstracts and full-text articles will be uploaded to Endnote and then imported to Covidence®. Prior to the formal screening process, a calibration exercise will be undertaken to pilot and refine the screening questions.

### Selection process

Three review authors (MM, AB, and CB) will independently screen all titles and abstracts identified from searches to determine which meet the inclusion criteria. We will obtain full reports for all titles that appear to meet the inclusion criteria or where there is any uncertainty. Three authors (MM, AB, and CB) will independently screen full-text articles for inclusion or exclusion, with discrepancies resolved by discussion and by consulting a third author if necessary, to reach consensus. We will seek additional information from study authors where necessary to resolve questions about eligibility. All potentially relevant papers excluded from the review at this stage will be listed as excluded studies, with reasons for exclusion recorded. We will also provide citation details and any available information about ongoing studies, and collate and report details of duplicate publications, so that each study (rather than each report) is the unit of interest in the review. We will report the screening and selection process in an adapted PRISMA flow chart [47]. Neither of the review authors will be blind to the journal titles or to the study authors or institutions.

### Data collection process

Using standardised forms developed using Microsoft® Excel spreadsheet software, two reviewers will extract data independently from each eligible study. To ensure consistency across reviewers, we will conduct calibration exercises prior to commencing the review. Data extracted will include demographic information, methodology, intervention details, and all reported outcomes (Additional file 6).

Reviewers will resolve disagreements by discussion, and an arbitrator (KW) will adjudicate unresolved disagreements. We will contact study authors to resolve any uncertainties.

We will search for multiple reports of a single study and report by study not publications. Articles deemed to be duplicates will be recorded and not included in the analysis.

### Risk of bias in included studies

To facilitate the assessment of possible risk of bias for each study, we will assess and report on the methodological risk of bias of included studies in accordance with the Cochrane Handbook [48]. We will use the Cochrane risk of bias (RoB) 2.0 tool for assessing the risk of bias for randomised controlled trials and the Risk of Bias in Non-Randomised Studies of Interventions (ROBINS-I) tool for non-randomised studies. A fixed set of bias domains is included in each of these tools, which are intended to cover all issues that might lead to a risk of bias [48]. We will judge each domain as being at ‘Low’ or ‘High’ risk of bias or ‘Some concerns’. Judgements about risk of bias will be supported by written justifications. Due to our expectation that there will be a limited number of randomised studies on this topic, we will include non-randomised before and after studies using the ROBINS-1 tool to assess the following biases: pre-intervention, at-intervention, and post-intervention features of the study. Studies will be judged as ‘Low’, ‘Moderate’, ‘Serious’, or ‘Critical’ risk of bias based on answers to the signalling questions.

In all cases, two authors will independently assess the risk of bias of included studies, with any disagreements resolved by discussion to reach consensus and then by consulting a third author for arbitration. We will contact study authors for additional information about the included studies, or for clarification of the study methods as required. We will incorporate the results of the risk of bias assessment into the review through standard tables, and systematic narrative description and commentary about each of the elements, leading to an overall assessment of the risk of bias of included studies and a judgement about the internal validity of the review’s results.

## Data synthesis

We will summarise the PICO characteristics of each study in a ‘Characteristics of included studies’ table using the following domains: setting, study design, participants, primary purpose of intervention, comparator, simulation scenario, simulation duration, evaluation instrument, follow-up, and significant findings. The resulting descriptions will be used to determine which studies can be grouped for synthesis.

The decision to meta-analyse data will be based on whether the interventions in the included trials are similar enough in terms of participants, settings, intervention, comparison, and outcome measures to ensure meaningful conclusions from a statistically pooled result. If studies are sufficiently homogenous in terms of design and comparator, we will conduct meta-analyses using a random-effects model.

If we are unable to pool the data statistically using meta-analysis, we will provide clear reasons for this decision and will conduct a narrative synthesis of results. We will present the major outcomes and results, organised by intervention categories according to the major types and/or aims of the identified interventions. Depending on the assembled research, we may also explore the possibility of organising the data by population. Within the data categories, we will explore the main comparisons of the review: (1) intervention versus no intervention, (2) intervention versus usual care, and (3) one form of intervention versus another. Where studies compare more than one intervention, we will compare each separately to no intervention/control and with one another.

### Assessment of heterogeneity

Where studies are considered similar enough, based on consideration of populations, interventions, or other factors, to allow pooling of data using meta-analysis, we will assess the degree of heterogeneity by visual inspection of forest plots and by examining the chi^2^ test for heterogeneity. We will report our reasons for deciding that studies were similar enough to pool statistically. Heterogeneity will be quantified using the *I*^2^ statistic. An *I*^2^ value of 50% or more will be considered to represent substantial levels of heterogeneity, but this value will be interpreted in light of the size and direction of effects and the strength of the evidence for heterogeneity, based on the *P* value from the chi^2^ test [48]. Where heterogeneity is present in pooled effect estimates, we will explore possible reasons by conducting subgroup analysis.

Where we detect substantial clinical, methodological, or statistical heterogeneity across included studies, we will not report pooled results from meta-analysis but will instead use a narrative approach to data synthesis. In this event, we will clearly report our reasons for deciding that studies were too dissimilar to meta-analyse. We will also attempt to explore possible clinical or methodological reasons for this variation by grouping studies that are similar in terms of populations, intervention features, and methodological features to explore differences in intervention effects.

### Meta-bias(es)

We will assess publication bias qualitatively based on the characteristics of the included studies (e.g. if only small studies that indicate positive findings are identified for inclusion), and if information that we obtain from contacting experts and authors or studies suggests that there are relevant unpublished studies. If we identify sufficient studies (at least 10) for inclusion in the review, we will construct a funnel plot to investigate small study effects, which may indicate the presence of publication bias. We will formally test for funnel plot asymmetry, with the choice of test based on advice in the Cochrane Handbook for Systematic Reviews on Interventions [48], and bearing in mind that there may be several reasons for funnel plot asymmetry when interpreting the results.

### Subgroup analysis and investigation of heterogeneity

Subgroup analyses will be used to explore possible sources of heterogeneity, based on the following: (1) participant characteristics, (2) intervention, and (3) follow-up period.

We will use the following outcomes in subgroup analysis: (1) participant characteristics (profession, health care specialty, initial or continuing training), (2) intervention (type of simulation, simulation alone or with adjunct pedagogy, disorder studied, frequency and length of simulation, educational purpose), and (3) follow-up (3, 6, and 12 months, 2 years post-training).

### Sensitivity analysis

We will perform sensitivity analyses defined a priori to assess the robustness of our conclusions and explore its impact on effect sizes. This will involve the following:

1. Restricting the analysis to published studies
2. Restricting the analysis to studies with a low risk of bias
3. Imputing missing data

### Measures of treatment effect

We will estimate the effect of the intervention using risk ratio/risk difference for dichotomous data, together with the appropriate associated 95% confidence interval, and mean difference or standardised mean difference for continuous data, together with the 95% appropriate associated confidence interval. We will ensure that an increase in scores for continuous outcomes can be interpreted in the same way for each outcome, explain the direction to the reader, and report where the directions were reversed if this was necessary. Skewed data and non-quantitative data will be presented descriptively.

### Unit of analysis issues

The primary analysis will be per individual randomised; however, all included trials will be assessed in order to determine the unit of randomisation and whether or not this unit of randomisation is consistent with the unit of analysis. Special issues in the analysis of studies with non-standard design, like cluster randomised trials, crossover trials, and studies with multiple treatment groups, will be addressed. For cluster randomised trials, we will extract an intraclass correlation co-efficient to modify the results according to the methods described in the Cochrane Handbook for Systematic Reviews of Interventions [48]. For crossover trials, a major concern is the carry-over effect. We will only use data from the first phase. When a study has more than two treatment groups, we will present the additional treatment arms. Where the additional treatment arms are not relevant, they will not be taken into account. We will also acknowledge heterogeneity in the randomisation unit and perform a sensitivity analysis.

### Dealing with missing data

We will attempt to contact study authors to obtain missing data (participant, outcome, or summary data). For participant data, we will, where possible, conduct analysis on an intention-to-treat basis; otherwise, data will be analysed as reported. We will report on the levels of loss to follow-up and assess this as a source of potential bias. For missing outcome or summary data, we will impute missing data where possible and report any assumptions in the review. We will investigate, through sensitivity analyses, the effects of any imputed data on pooled effect estimates.

### Confidence in cumulative evidence

We will prepare a ‘summary of findings’ table to present the results of meta-analysis and/or narrative synthesis for each primary outcome of this review. We will use the GRADE approach (Grading of Recommendations Assessment, Development, and Evaluation) for assessing certainty (or quality) of the body of evidence for a given outcome.

The quality of evidence will be assessed across the domains of risk of bias, consistency, directness, precision, and publication bias. Additional domains may be considered where appropriate. Quality will be adjudicated as high (further research is unlikely to change our confidence in the estimate of effect), moderate (further research is likely to have an important impact on our confidence in the estimate of effect and may change the estimate), low (further research is very likely to have an important impact on our confidence in the estimate of effect and is likely to change the estimate), or very low (very uncertain about the estimate of effect). If meta-analysis is not possible, we will present results in a narrative ‘summary of findings’ table format.

## Discussion

We anticipate the initial search yield for this study will be large as training interventions which include role plays could be classified as simulated experiences. The two main potential challenges include deciding at a study level which training formats can be classified as simulation and if simulation-based education which focusses on working with patients with mental health issues or psychiatric illness is primarily focussed on managing aggression.

An advisory group, including hospital aggression response (code grey) team coordinators, hospital aggression response (code grey) team members, simulation faculty staff and health care workers who understake management of aggression training, will be formed [48].

### Amendments to protocol

The proposed systematic review will be reported in accordance with the reporting guidance provided in the Preferred Reporting Items for Systematic Reviews and Meta-analyses (PRISMA) statement [43]. Any amendments made to this protocol during the study conduct will be reported in the final manuscript and reported in PROSPERO.

### Strengths and limitations

The strength of this review is the comprehensive review of training in this specific but increasingly common acute health care issue. Acute care hospital staff are not routinely trained to manage aggression however are expected to use highly developed communication skills to effectively de-escalate and intervene whilst not causing patient harm. The results of this review may assist in the design and development of training programmes for staff working in acute care settings who are exposed to aggression and other externalising behaviours.

We anticipate that publication bias and heterogeneity may pose a limitation for this review. There is also the possibility of reporting bias. In addition, this study is restricted to published reports in the English language.

We will attempt to mitigate these issues by highlighting decision criteria, limitations, and bias and using caution in the interpretation of findings.

### Dissemination

The findings of this review will be disseminated through publication in a peer-reviewed journal and presentation at relevant conferences.

This systematic review will provide relevant evidence about the effectiveness of simulation-based education utilising simulated patients in teaching management of clinical aggression to health care providers working in the acute health care setting.

If simulation is effective, then these methods should be implemented to train health care professionals working with hospitalised children, young people, and adults who display aggression in the clinical setting. If simulation is not effective or if there is insufficient evidence, innovation and more high-quality studies may be needed.

## Supplementary information

**Additional file 1.** PRISMA-P 2015 Checklist.

**Additional file 2.** Different forms of simulation evaluated in this review.

**Additional file 3.** Terms used in search strategy for aggressive or externalising behaviours.

**Additional file 4.** Definitions of outcome measures.

**Additional file 5.** MEDLINE search strategy (draft).

**Additional file 6.** Data items.

## Data Availability

The datasets generated by this systematic review will be available from the corresponding author on reasonable request.

## Abbreviations

ASD: Autism spectrum disorder
