# Supplementary material for: Simulation-based education for teaching aggression management skills to health care providers in the acute health care setting: a systematic review protocol
Source: Syst Rev. 2020 Sep 4;9:208. doi: 10.1186/s13643-020-01466-8 (PMC7487524; doi:10.1186/s13643-020-01466-8)
Supplement: Supplementary file 2 — Additional file 2. Different forms of simulation evaluated in this review. [file 13643_2020_1466_MOESM2_ESM.docx]

**Additional File 2: Different forms of simulation evaluated in this review (1)**

| **Simulation types using people as patients** | **Definition** |
| --- | --- |
| Standardised (StP) or simulated patient (SP) | An individual trained to portray a patient with a specific condition in a realistic, standardized, and repeatable way and where portrayal/presentation varies based only on learner performance; this strict standardization of performance in a simulated session is what can distinguish standardized patients from simulated patients. |
| Simulated patient | A person who has been carefully coached to simulate an actual patient so accurately that the simulation cannot be detected by a skilled clinician. In performing the simulation, the SP presents the gestalt of the patient being simulated; not just the history, but the body language, the physical findings, and the emotional and personality characteristics as well (Barrows, 1987). Often used interchangeably with standardized patients in the USA and Canada, but in other countries simulated patient is considered a broader term than standardized patient, because the simulated patient scenario can be designed to vary the SP role in order to meet the needs of the learner. |
| Standarised patient simulation | A simulation using a person or persons trained to portray a patient scenario or actual patient(s) for health care education (Society for Simulation in Healthcare). |
| Simulationist | An individual who is involved in the design, implementation, and/or delivery of simulation activities; for example, educators, technologists, operations specialists, technicians (Society for Simulation in Healthcare]. |
| Role player | One who assumes the attitudes, actions, and discourse of (another), especially in a make-believe situation, in an effort to understand a differing point of view or social interaction. For example: Nursing students were given a chance to role play a patient or a surgeon. This term is sometimes used interchangeably with the terms ‘simulated’ and ‘standardized patient’ and may include medical, nursing, or other health professionals. (Victorian Simulated Patient Network). See also: ACTOR, CONFEDERATE, EMBEDDED PARTICIPANT, SIMULATED PATIENT, SIMULATED PERSON, STANDARDIZED PATIENT. |
| Simulation Fidelity | The physical, semantic, emotional, and experiential accuracy that allows persons to experience a simulation as if they were operating in an actual activity (Society for Simulation in Healthcare). |

**Reference:**

1. Lopreiato JO. Healthcare Simulation Dictionary. Rockville, MD: Agency for Healthcare Research and Quality; 2016.
