# Supplementary material for: Simulation-based education for teaching aggression management skills to health care providers in the acute health care setting: a systematic review protocol
Source: Syst Rev. 2020 Sep 4;9:208. doi: 10.1186/s13643-020-01466-8 (PMC7487524; doi:10.1186/s13643-020-01466-8)
Supplement: Supplementary file 3 — Additional file 3. Terms used in search strategy for aggressive or externalising behaviours. [file 13643_2020_1466_MOESM3_ESM.docx]

**Additional File 3: Terms used in search strategy for aggressive or externalising behaviours**

| **Search terms** |
| --- |
| Challenging behaviour  Problem behaviour  Behavioural problems  Aggression  Self-injury  Self-injurious behaviour  Self-harm  Behaviours of concern  Workplace violence  Occupational violence  Assaultive behaviour  Hostility  Irritable mood  Anger  Self-stimulating behaviour  Abusive behaviour  Agitation  Agonistic behaviour  Dangerous behaviour  Destructive behaviour  Combative behaviour  Disruptive behaviour  Attacking behaviour  Rage  Threatening behaviour  Impulsive behaviour  Acting out |
