# Supplementary material for: Simulation-based education for teaching aggression management skills to health care providers in the acute health care setting: a systematic review protocol
Source: Syst Rev. 2020 Sep 4;9:208. doi: 10.1186/s13643-020-01466-8 (PMC7487524; doi:10.1186/s13643-020-01466-8)
Supplement: Supplementary file 4 — Additional file 4. Definitions of outcome measures. [file 13643_2020_1466_MOESM4_ESM.docx]

**Additional File 4: Definitions of outcome measures**

| **Outcome measure** | **Definition** |
| --- | --- |
| Aggression | Forceful physical, verbal or symbolic action which is either appropriate, self-protective, or inappropriate (1) |
| Frequency of clinical aggression | Frequency of episodes of aggression which occurs from patients in a health care setting |
| Frequency / number of behavioural emergency situations | Frequency of behaviour emergencies documented in the health care agency. |
| Physical restraint | The use of restraint to hands, upper and or lower limbs to forcibly control a patient (2) |
| Chemical restraint | A sedative or tranquilizer given to a patient to reduce agitation or hazardous behaviour (3) |
| Mechanical restraint | A device used on a person to restrict free movement (4) |
| Patient/ family complaints | Documented complaints about care provided to patient and or family |
| Patient harm | Anything that impairs or adversely affects the safety of a patient (5) |
| Staff harm | Anything that impairs or adversely affects the safety of staff (5) |
| Activation of emergency response | Activation or utilisation of the hospital emergency response system |
| Utilisation of skill taught in simulation | Demonstrated use of skills that were taught and or discussed in the simulation training program |
| Workload | The amount of work provided by a health professional (6) |
| Work morale | The state of the spirit, confidence or willingness to perform assigned tasks of individuals or a group when at work (7) |

**References:**

1. Segen's Medical Dictionary, Farlex Inc. 2011. In: The Free Dictionary [Intranet] [Internet]. Available from: <https://medical-dictionary.thefreedictionary.com/aggression>.

2. Miller-Keane Encyclopedia and Dictionary of Medicine, Nursing and Allied Health. 2003. In: The Free Dictionary [Internet] [Internet]. Saunders, an imprint of Elsevier Inc. 7th. Available from: <https://medical-dictionary.thefreedictionary.com/physical+restraint>.

3. Segen's Medical Dictionary, Farlex Inc. 2011. In: The Free Dictionary [Internet] [Internet]. Available from: <https://medical-dictionary.thefreedictionary.com/chemical+restraint>.

4. McGraw-Hill Concise Dictionary of Modern Medicine. 2002. In: The Free Dictionary [Internet] [Internet]. The McGraw-Hill Companies Inc. Available from: <https://medical-dictionary.thefreedictionary.com/mechanical+restraint>.

5. Medical Dictoinary, Farlex and Partners. 2009. In: The Free Dictionary [Internet] [Internet]. Available from: <https://medical-dictionary.thefreedictionary.com/harm>.

6. Segen's Medical Dictionary. 2011. In: The Free Dictionary [Internet] [Internet]. Available from: <https://medical-dictionary.thefreedictionary.com/workload>.

7. American Heritage Dictionary of the English Language. 2011. In: The Free Dictionary [Intranet] [Internet]. 5th. Available from: <https://www.thefreedictionary.com/morale>.
