# Supplementary material for: Simulation-based education for teaching aggression management skills to health care providers in the acute health care setting: a systematic review protocol
Source: Syst Rev. 2020 Sep 4;9:208. doi: 10.1186/s13643-020-01466-8 (PMC7487524; doi:10.1186/s13643-020-01466-8)
Supplement: Supplementary file 5 — Additional file 5. MEDLINE search strategy (draft). [file 13643_2020_1466_MOESM5_ESM.docx]

**Additional File 5: MEDLINE search strategy (draft)**

Database(s): **Ovid MEDLINE(R) ALL** January 1980 onwards
Search Strategy:

| **#** | **Searches** |
| --- | --- |
| 1 | *Role Playing/ |
| 2 | (role adj play*).tw,kf. |
| 3 | exp *simulation training/ |
| 4 | simulation.tw,kf. |
| 5 | *communication/ or *nonverbal communication/ or *teach-back communication/ or exp *verbal behavior/ |
| 6 | (communication or communicate).tw,kf. |
| 7 | (non-technical adj skill*1).tw,kf. |
| 8 | exp *Aggression/ |
| 9 | exp *professional-patient relations/ |
| 10 | *workplace violence/ |
| 11 | *Clinical Competence/ |
| 12 | (team* or (situation adj awareness) or (human adj factor*)).tw,kf. |
| 13 | (nurse* or nursing or doctor* or physician* or specialist* or (allied adj health*) or resident* or fellow* or trainee* or therapist*).tw,kf,hw. |
| 14 | exp *Health Personnel/ |
| 15 | (hospital* or tertiary or acute-care).tw,kf,hw. |
| 16 | ((Challenging or problem* or a?saultive or abusive or agonistic or dangerous or combative or attacking or threatening or impulsive) adj3 behavio?r*).tw,kf. |
| 17 | exp *Self-Injurious Behavior/ |
| 18 | ((Destructive or distruptive) adj3 behavio?r*).tw,kf. |
| 19 | *Hostility/ |
| 20 | *Irritable mood/ |
| 21 | exp *Anger/ |
| 22 | (self-stimulat* adj3 behavio?r*).tw,kf. |
| 23 | Behavio?r* of concern.tw,kf. |
| 24 | (De-escalation or deescalation).tw,kf. |
| 25 | ((Severe adj tantrum*) or self-harm or self-stimulat* or agitation or rage or acting-out).tw,kf. |
| 26 | ((Occupational or workplace or work-place) adj violence).tw,kf. |
| 27 | (1 or 2 or 3 or 4) and (5 or 6 or 7 or 8 or 9 or 10 or 11 or 12 or 16 or 17 or 18 or 19 or 20 or 21 or 22 or 23 or 24 or 25 or 26) and ((13 or 14) and 15) |

*no limits were used within this search
