# Supplementary material for: Simulation-based education for teaching aggression management skills to health care providers in the acute health care setting: a systematic review protocol
Source: Syst Rev. 2020 Sep 4;9:208. doi: 10.1186/s13643-020-01466-8 (PMC7487524; doi:10.1186/s13643-020-01466-8)
Supplement: Supplementary file 6 — Additional file 6. Data items. [file 13643_2020_1466_MOESM6_ESM.docx]

**Additional File 6: Data items**

| **Study Details** | Authors  Journal  Year of Publication  Year recruitment began  Country |
| --- | --- |
| **Study Objectives** |  |
| **Participant Characteristics** | Profession  Role  Years of clinical experience  Gender  Previous training – simulation  Previous training – clinical aggression  Numbers of participants |
| **Methods** | Design  Allocation  Sampling  Blinding  Data collection time points  Loss to follow-up  Recruitment rates  Retention rates  Comparison/ control group  Risk of bias |
| **Intervention** | Training:   - Simulation only - Simulation + web resources - Simulation + lecture/workshop - Simulation + lecture/workshop + web resources   Setting:   - Location of training - Environment simulated   Description:   - Recruitment for SP - Type of simulation (role play, simulated patient (SP)) - Simulation alone or with adjunct training/resources - Description of adjunct training - Scenario summary - Clinical variations - Range of task difficulty - Duration of each simulation - Number of simulation sessions completed - Educational purpose of program - Feedback – individual, group |
| **Outcomes** | Description of main findings  Effects of intervention on: (Kirkpatrick ranking, measurement instruments, evaluator, unit of measurement, timing of assessment   - Changes in knowledge - Changes in skill - Change in confidence - Change in self-perceived competence - Staff attitudes to aggression - Satisfaction with learning experience - Patients – experience, emergency situations, use of restraint |
